# Multi-Walled Carbon Nanotubes Functionalized with Hydroxamic Acid Derivatives for the Removal of Lead from Wastewater: Kinetics, Isotherm, and Thermodynamic Studies

**DOI:** 10.3390/polym14183870

**Published:** 2022-09-16

**Authors:** Yasair S. S. Al-Faiyz, Mohamed Gouda

**Affiliations:** Department of Chemistry, Faculty of Science, King Faisal University, P.O. Box 380, Al-Ahsa 31982, Saudi Arabia

**Keywords:** MWCNTs, lead removal, hydroxamic acids, nanocomposites, wastewater treatment

## Abstract

Hydroxamic acids are recognized chelators for various metals; however, using them as functional groups on carbon nanotubes (CNTs) is rare. In this study, novel multi-walled carbon nanotubes (MWCNTs) functionalized with hydroxamic acid derivatives were developed. The MWCNTs were first oxidized, and the resulting product, MWCNT-COOH (A), was treated with oxalyl chloride to yield MWCNT-COCl. The functionalized MWCNTs were susceptible to reacting with the hydroxylamine derivatives of type R–NHOH and produced MWCNTs functionalized with the following hydroxamic acid derivatives (MWCNT-HA): MWCNT-CONOHMe (B), MWCNT-CONOHCOMe(C), and MWCNT-CONOHPh (D). The synthesized derivatives were confirmed by various techniques such as scanning electron microscopy, X-ray photoelectron spectroscopy, and Raman spectroscopy. In order to examine their chelation ability, these materials were examined as possible new adsorbents for harmful Pb(II) particles. The adsorption efficiency of the functionalized MWCNT adsorbents toward Pb(II) was investigated. The effects of the adsorbent dose, temperature, pH, and time on adsorption efficiency were considered, and adsorption boundaries that resulted in enhanced effectiveness were obtained. The developed materials were found to have extraordinary coordination sites, such as amine, hydroxyl, and carboxyl groups, which served as excellent chelating specialists for the Pb(II) particles. Thermodynamic and kinetic investigations revealed the unconstrained nature of the adsorption of Pb(II) by the developed MWCNT adsorbents at room temperature. The adsorption was noted to follow the pseudo-second-order and Langmuir isotherm models.

## 1. Introduction

Allotropes of carbon with nanostructures that have a length-to-diameter ratio higher than 1,000,000 are known as carbon nanotubes (CNTs). These cylindrical carbon molecules have unique features that might make them valuable in a variety of nanotechnology applications [1]. In terms of tensile strength and elastic modulus, respectively, carbon nanotubes are the stiffest and strongest materials that have yet to be identified. The covalent sp^2^ bonds that form between the various carbon atoms are what provide the compound with its strength. C-C bonds are predicted to make CNTs incredibly strong along their axes and provide them with an extraordinarily high Young’s modulus in their axial direction [2]. Carbon nanotubes have fascinating electrical characteristics in addition to being highly strong. In comparison to metals such as copper, metallic nanotubes have a theoretical electrical current density of 4109 A/cm^2^, which is more than 1000 times higher [3]. All nanotubes are anticipated to have excellent thermal insulators laterally to the tube axis and extremely good thermal conductors down the tube, displaying a characteristic known as “ballistic conduction” [4,5]. Because of the curvature of the CNT surface, its chemical reactivity is increased when compared to a graphene sheet. The mixing of the orbitals due to this curvature results in the hybridization of the orbitals [6]. Since chiral nanotubes lose their optical activity as they get larger, it is hypothesized that these factors can also affect other physical characteristics. CNTs may play a significant role in optical devices made using optical activity [7].

**Figure 1:**
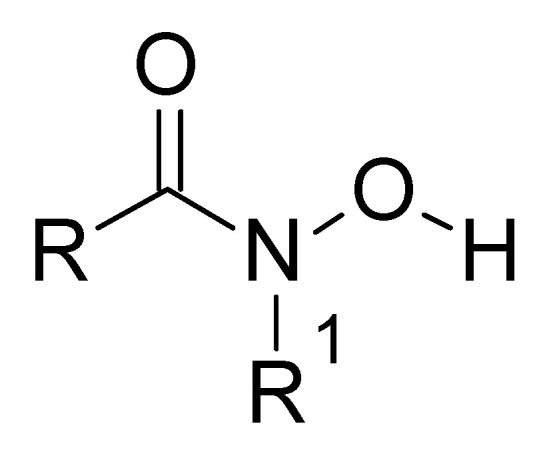
Hydroxamic acid, with R and R^1^ as organic residues and CO as a carbonyl group. They are amides (RC(O)NHR’), wherein the NH center has an OH substitution.

Carbon nanotubes (CNTs) have attracted scientific attention in various fields because of their chemical, thermal, and structural properties [8]. Functionalized CNTs play a significant role in medicine [9], engineering [10,11], and industrial applications [12]. The functionalization of CNTs is known to enrich their physical and chemical properties [13]. Therefore, various functional groups have been introduced, and several chemical approaches have been investigated in this regard [8,9,10,11,12,13]. For instance, CNTs were functionalized with the carboxylic group through Cage-Opening Buckminster-fullerene and refluxing with hydroxylamine to enhance the luminescence properties of fullerene C_60_ [9]. Furthermore, porous nanocomposite scaffold hydroxamic-CNT was prepared with a combination of a hydroxamic derivative of alginate and an amine-functionalized multi-walled carbon nanotube (CNT) [14]. In addition, alginate–chitosan/MWCNTs as a novel support for Ag_2_O immobilization have been prepared and used in a catalytic reduction of 4-nitrophenol as an example of water pollutants [15]. Moreover, CNTs have been functionalized with hydroxamic acid by binding them strongly to basic metal oxide surfaces and have been applied in field-effect transistors [16]. However, less attention has been paid to hydroxamic-functionalized CNTs [16,17,18]. Hydroxamic acids have an extremely rich reactive chemistry as shown in Figure 1, and the ability to react with metals, which has led to them being extensively applied in various fields. One of their most important applications involves the removal of toxic metals [19].

Therefore, the functionalization of multi-walled carbon nanotubes (MWCNTs) with hydroxamic acid derivatives was investigated in this study, and their application in the removal of lead from wastewater was examined. The contamination of water via metals is a significant concern because certain metals are exceptionally harmful even at low concentrations [20]. The harmfulness of metal contaminants is unavoidable if they gather in the delicate tissues of the human body [21,22]. These metals can enter the human body via many routes. One of these routes involves potable water; moreover, unlike natural toxins, these metal ions at substantial concentrations are non-biodegradable. Heavy metal particles exist in wastes produced by various industries such as leather tanning, metal plating, radiator fabrication, battery production, mining, and fertilizer enterprises [23]. Lead is known to be a significantly poisonous metal and can infiltrate the human body through ingestion, inward breathing, or through the skin [24,25]. Once inside the body, lead is ingested and stored in bones, blood, and tissues [26]. Lead can cause extreme harm to the brain and kidneys and can eventually be fatal. By recreating calcium, lead can cross the blood–brain barrier and annihilate the myelin sheaths of neurons, diminishing their numbers; restrain neurotransmission courses; and inhibit the development of neurons [27]. Numerous powerful adsorbents have been developed in this regard. For instance, a new hybrid adsorbent called PANI@Fe-Mn-Zr was created by combining polyaniline (PANI) and the Fe-Mn-Zr metal oxide composite [28]. It was used to remove the dye methyl red from aqueous solutions. Additionally, a brand-new nanocomposite made of PANI and magnesium ferrite (MgFe_2_O_4_-PANI-NC) was effectively prepared and used for methyl red dye removal from water [29]. The efficiency of magnesium ferrite nanoparticles (MgF-NPs) as an adsorbent for the removal of hazardous malachite green dye from an aqueous solution was assessed under ultrasonic irradiation [30]. The salicylic hydroxamic acid catalyst used to reduce the chromaticity of effluent from a simulated mineral processing plant using ozonation catalyzed by granular activated carbon was investigated [31]. How to make environmentally friendly cellulose acetate/graphene oxide nanocomposite adsorbents for usage in extracting Ni^2+^ from wastewater using environmental applications was investigated [32]. The flexible membrane ingredients of vinyl–imidazolium-based polyelectrolyte composites were structurally improved by being soaked with multivalent imidazolium–benzene ionic liquids or crosslinked with novel cationic crosslinkers that have interior imidazolium cations and vinyl–imidazolium cations at the border to aid the discrimination and simplification of CO_2_ absorption through the film [33].

MWCNTs are among the best metal adsorbents and have been used in numerous commercial applications [34].

A few studies have revealed that CNTs are outstanding in terms of water treatment and the adsorption of heavy metals, such as copper, lead, and cadmium. CNTs have also shown high productivity in polar and nonpolar natural particles and organic and biological pollutants [35,36,37]. These remarkable capabilities make them a good choice for water treatment and water desalination [35,38,39]. This is attributed to the various physical and chemical interactions between contamination and MWCNTs facilitated by π–π interactions, covalent bonding, hydrogen bonding, [39] and electrostatic connections [40]. In addition, their surfaces are composed of multiple layers of graphene sheets, which provide available spaces for pollutants to adsorb into them [41,42]. Generally, they are highly sensitive toward contamination, antifouling, self-cleaning, reusable, and have good water permeability [35,43,44]. The functionalization of MWCNTs with P-, O-, and N-containing groups on their surfaces facilitates the control of surface territory, surface charge, hydrophobicity/hydrophilicity, and scattering. Furthermore, the adsorption limit and selectivity toward heavy metals can be improved [45,46]. For example, Li et al. [43] demonstrated the enhanced capacity of acid-refluxed CNTs to adsorb Pb(II) particles from water.

Moreover, Wang et al. [44] reported an increase in the zeta potential of oxidized MWCNTs, which resulted in the development of a negative surface charge because of the creation and ionization of useful groups (-COOH and OH). The adsorption of Pb(II) onto acidic MWCNTs was noted to be nonuniform, with the Pb(II) ions preferentially gathering on the capped and blemished sites of the MWCNTs. Recent attention has been paid to attractive adsorbents to avoid issues related to adsorption recovery. The fundamental preferences of these attractive composite adsorbents include high strength, excellent adsorption rate, and improved adsorption capacity [47,48]. Hydroxamic acids are well known as chelators for the absorption of various metals; however, using them as functional groups on CNTs is rare.

By decontaminating polluted wastewater utilizing low-cost adsorbents, one can obtain the benefits of water treatment. Herein, MWCNTs were functionalized with novel hydroxamic acid derivatives utilizing simple methods. Following that, the impacts of various adsorption process parameters on the removal of harmful metals such as Pb(II) from polluted water onto synthesized materials were investigated and optimized. Furthermore, the adsorption kinetics, isotherms, thermodynamics, and removal capacity of the synthesized materials were examined using a variety of adsorption investigations.

## 2. Materials and Methods

### 2.1. Materials

The fine chemicals used in the experiments were purchased from Sigma-Aldrich Chemie GmbH, (Taufkirchen, Germany). All solvents used in experiments were HPLC grade and used as received without further purification. MWCNTs were purchased from Chengdu Organic Chemicals Co, Ltd., (Chines Academy of Sciences, Chengdu, China). The MWCNTs had the following technical details: Graphitized MWCNTs-COOH, -COOH content: 0.25 wt. %, 0D: >50 nm, length: 10–20 um, purity: 99.9%.

### 2.2. Functionalization of MWCNTs

**Scheme 1:**
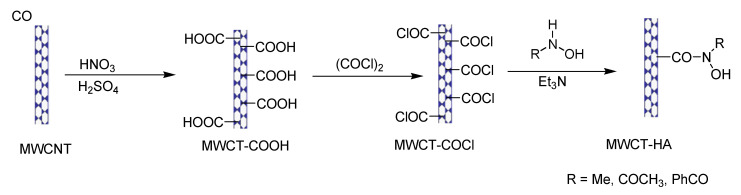
Proposed steps for the synthesis of multi-walled carbon nanotubes (MWCNTs) functionalized using hydroxamic acid derivatives.

MWCNTs were first oxidized using concentrated nitric acid and sulfuric acid in a sonicated vessel to yield MWCNT-COOH and subsequently treated with oxalyl chloride to convert the carboxylic group into an acid chloride group (MWCNT-COCl). The prepared MWCNT-COCl was susceptible to reacting with hydroxylamine derivatives of type R–NHOH and produced functionalized MWCNTs with hydroxamic acid derivatives. The synthesis steps are detailed henceforth. First, the MWCNTs (0.5 g) were refluxed with 100 mL of 70% HNO_3_ for 24 h and subsequently washed with 300 mL of deionized water until the pH of the filtrate was roughly equal to 7. The produced MWCNT-COOH (denoted as A) was filtered and dried in a vacuum at 55 °C for 24 h. In the second step, MWCNT-COOH (0.1 g) was refluxed with an excess of oxalyl chloride using an ultrasonic bath for 24 h at 65 °C to form MWCNT-COCl. In the third step, triethyl amine (Et_3_N; 1_eq_) was added to a solution of 20.8 mmol of readily available hydroxyl amine derivatives (R–NHOH, where R = –CH_3_, –COCH_3_, and PhCO–), followed by dropwise addition of MWCNT-COCl at 0 °C for 6 h and subsequent stirring at room temperature for 6 h. The reaction mixture was subsequently filtered and washed with excess deionized water. The resulting black solid was dried under a vacuum, and the obtained MWCNT-HA derivatives were labeled as MWCNT-CON(OH)CH_3_ (B), MWCNT-CON(OH)COCH_3_ (C), and MWCNT-CON(OH)COPh (D). The synthesis steps are shown in Scheme 1.

### 2.3. Characterization of Prepared MWCNTs

The MWCNTs functionalized with different hydroxamic acid derivatives were characterized using different analytical methods, such as Fourier transform infrared spectroscopy (FTIR), which was performed using an FTIR spectrometer (Cary 630, Agilent Technologies, Penang, Malaysia). The spectra were analyzed within the range of 4000–400 cm^−1^. The surface morphologies of the prepared MWCNT–hydroxamic acid derivatives were examined by field emission scanning electron microscopy (FESEM) using an electron probe microanalyzer (JXA-840, JOEL, Tokyo, Japan). Raman spectra were recorded using an XploRA PLUS instrument (Horiba, Japan). The functional groups on the surface of the MWCNTs were examined using X-ray photoelectron spectroscopy (XPS; ESCALAB Xi, Thermo Fisher Scientific, Waltham, MA, USA). The concentration of PB(II) was determined by atomic absorption spectroscopy (VarianSpectrAA 220, Spectralab, Toronto, CA, USA).

### 2.4. Removal of Heavy Metal Ions

The adsorbents, MWCNT-COOH (A), MWCNT-CON(OH)CH_3_ (B), MWCNT-CON(OH)COCH_3_ (C), and MWCNT-CON(OH)COPh (D), were utilized to eliminate Pb(II) ions from aqueous solutions. MWCNT-COOH (A), MWCNT-CON(OH)CH_3_ (B), MWCNT-CON(OH)COCH_3_ (C), and MWCNT-CON(OH)COPh (D), (0.1 g each) were impregnated in 20 mL solutions of Pb(II) at various concentrations (10–35 mg/L) and at different pH ranges (1–12). The prepared solutions were mixed for various contact durations (10–120 min) at several temperatures (25–65 °C). Pb(II) adsorbed at equilibrium (mg g^−1^) was separated using a centrifuge at 3000 rpm and calculated using the following equation:

(1) $$q_{e}=\frac{\left(C_{0}-C_{e}\right)}{m}\times V$$

where *q_e_* is the lead adsorbed at equilibrium (mg g^−1^). *C*_0_, *C_t_*, and *C_e_* (ppm = mg L^−1^) represent the concentrations of lead at the initial time, time *t*, and the time of equilibrium, respectively; *V* is the volume of the lead solution (L), and *m* is the adsorbent weight (g) [46].

The removal efficiency (*R*%) was calculated as follows:

(2) $$R\%=\left(\frac{C_{0}-C_{t}}{C_{0}}\right)\times 100$$

where *C*_0_, *C_t_*, and *C_e_* represent the concentrations of lead at the initial time, time *t*, and the time at equilibrium, respectively.

The distribution coefficient (*K_d_*) of Pb(II) on the prepared adsorbent was calculated according to the following equation:

(3) $$K_{d}=\frac{q_{e}}{C_{e}}$$

where the distribution coefficient (*K_d_*) was used to assess the actual performance of the adsorbent [49,50]. All of the experiments were repeated 3 times, and the average of the results was recorded. The standard deviation (±SD) was calculated for all experiments.

#### 2.4.1. Kinetics of Adsorption

The adsorbate uptake rate was represented via kinetics analysis. Therefore, the mechanism of Pb(II) adsorption on the functionalized MWCNTs was elucidated by kinetic investigations. Pseudo-first-order and pseudo-second-order models were employed to explain the adsorption kinetics.

(4) $$q_{t}=q_{e}\left[1-\exp(-k_{1}t)\right]$$

(5) $$q_{t}=\left(\frac{k_{2}q_{e}^{2}t}{1+k_{2}q_{e}^{}t}\right)\times 100$$

where *k*_1_ and *k*_2_ are the rate constants of the pseudo-first-order and pseudo-second-order adsorption, respectively. This is typically expressed as the following equation:

(6) $$\ln(q_{e}-q_{t})=\ln q_{e}-k_{1}t$$

where *q_e_* is the amount of metal adsorbed per gram of the functionalized MWCNTs (mg g^−1^), *q_t_* is the amount of metal adsorbed at time *t* per gram of the functionalized MWCNTs (mg g^−1^), and *k*_1_ (min^−1^) is the pseudo-first-order adsorption rate constant. The pseudo-second-order kinetic model is dependent on the amount of solute adsorbed on the surface of the functionalized MWCNTs and the amount of Pb(II) adsorbed at equilibrium.

#### 2.4.2. Adsorption Isotherms

The mechanisms of interaction between the adsorbed metal and functionalized MWCNTs were examined by using various adsorption isotherm models. Experimental data fit well with the Langmuir and Freundlich models [51,52]. The Langmuir model considers the advancement of a monolayer of adsorbate on the normal surface of an adsorbent and is expressed as follows:

(7) $$\frac{C_{e}}{q_{e}}=\frac{1}{K_{L}q_{m}}+\frac{C_{e}}{q_{m}}$$

where *C_e_* is the adsorbate concentration at equilibrium (mmol L^−1^), *q_e_* is the amount of adsorbate adsorbed per unit weight of adsorbent (mmol g^−1^), *q_m_* is the adsorption capacity (mmol g^−1^) or monolayer capacity, and *K*_L_ is a constant (L mmol^−1^) [51,52]. The separation factor (*R_L_*) is a dimensionless number and is a fundamental characteristic of the Langmuir model. The equation for *R_L_* is expressed as follows:

(8) $$R_{L}=\frac{1}{1+k_{L}C_{o}}$$

where *C_o_* (mg L^−1^) is the highest initial concentration of the adsorbate and *K_L_* is the Langmuir constant (L mg^−1^). The value of *R*_L_ indicates the form of the isotherm to be either unfavorable (*R_L_* > 1), linear (*R_L_* = 1), favorable (0 < *R_L_* < 1), or irreversible (*R_L_* = 0). On the other hand, the Freundlich isotherm considers the adsorption between adsorbates and adsorbents with a heterogeneous surface. The rate of adsorption differs, as indicated by the energy level at the adsorption sites. Freundlich isotherms are obtained using the following equation:

(9) $$\ln q_{e}=\ln K_{f}+\frac{1}{n\ln C_{e}}$$

where *K_f_* (mmol/g) and 1/*n* are the constants of the system [52], *K_f_* is an indicator of the adsorption capacity of the adsorbent, and 1/*n* indicates the favorability of the adsorption process. The adsorption is favorable if 0 < *n* < 10. Langmuir and Freundlich isotherms can be used to obtain relationships between the amount of Pb(II) adsorbed on the MWCNT-COOH (A), MWCNT-CON(OH)CH_3_)(B), MWCNT-CON(OH)COCH_3_(C), and MWCNT-CON(OH)COPh(D) adsorbents and their equilibrium concentrations in solutions.

#### 2.4.3. Adsorption Thermodynamics

Experiments for examining the thermodynamics of adsorption were performed with a Pb(II) concentration of 100 mg L^−1^ at 298 K. The kinetic studies and mechanism of the adsorption process were investigated at different times. The pH value was fixed at 8 to avoid interaction with the hydroxyl group, adsorption time was varied from 1 to 30 min, and the adsorbent dose was 100 mg L^−1^.

The thermodynamic behavior was determined using the following equations [51,52]:

(10) $$K_{D}=\frac{q_{e}}{C_{e}}$$

(11) $$\Delta G=-RT\ln K_{D}$$

(12) $$\ln K_{D}=\left(\frac{\Delta S}{R}\right)-\left(\frac{\Delta H}{R}\right)\frac{1}{T}$$

where *K_D_* is the parcel constant at equilibrium; Δ*G* (kJ/mol) is the change in Gibbs free energy; *R* (8.314 J/mol K) is the gas constant; *T* (*K*) is the temperature; Δ*H* (kJ/mol) is the change in enthalpy; and Δ*S* (kJ/mol K) is the change in entropy. The Δ*G* values were determined using the *K_D_* estimates for each temperature, and the Δ*H* and Δ*S* values were determined using the slope and intercept of the plot of ln*K_D_* versus 1/*T*, respectively.

## 3. Results and Discussion

### 3.1. Scanning Electron Microscopy

**Figure 2:**
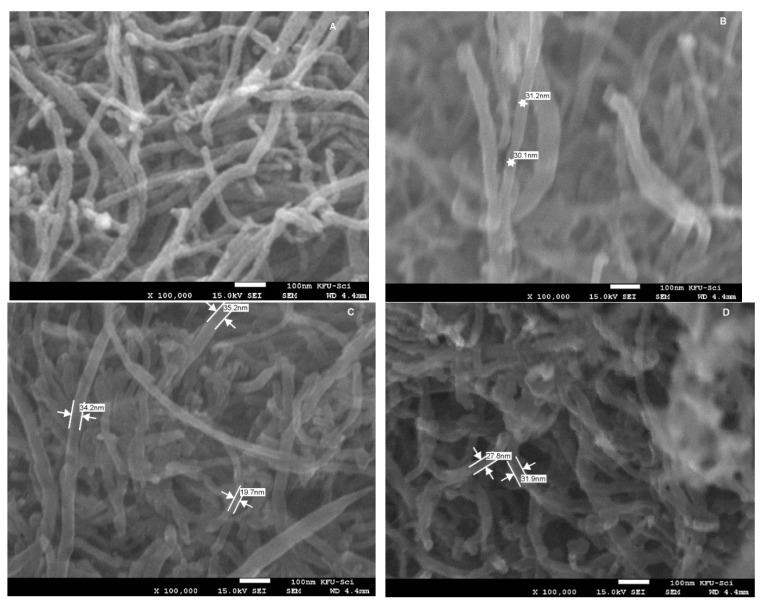
Scanning electron microscopy (SEM) images of (**A**) MWCNT-COOH, (**B**) MWCNT-CONOHMe, (**C**) MWCNT-CONOHCOMe, and (**D**) MWCNT-CONOHPh.

Figure 2 shows images obtained via scanning electron microscopy (SEM), which indicate the presence of an intricate network in all the samples. SEM was utilized to explore possible MWCNT fractures that occurred during treatment and used to distinguish between the morphological changes in the MWCNT samples based on the treatment. Figure 2A shows an SEM image of the oxidized MWCNTs with a mixture of H_2_SO_4_ and HNO_3_ causing serious carving on the graphitic surface of the material, prompting containers of the more limited length with numerous disarranged destinations [53,54]. Similar structures are visible in the images of MWCNTs treated with hydroxamic acid derivatives, MWCNT-CONOHMe (Figure 2B), MWCNT-CONOHCOMe (Figure 2C), and MWCNT-CONOHPh (Figure 2D). Notably, the underlying MWCNT structure in these composite MWCNTs was not harmed. The investigation of a few SEM images of the treated MWCNTs facilitated the measurement of the solid length of a couple of nanotubes, and all of them were in the range of 19–31 nm [55].

### 3.2. Raman Spectroscopy

**Figure 3:**
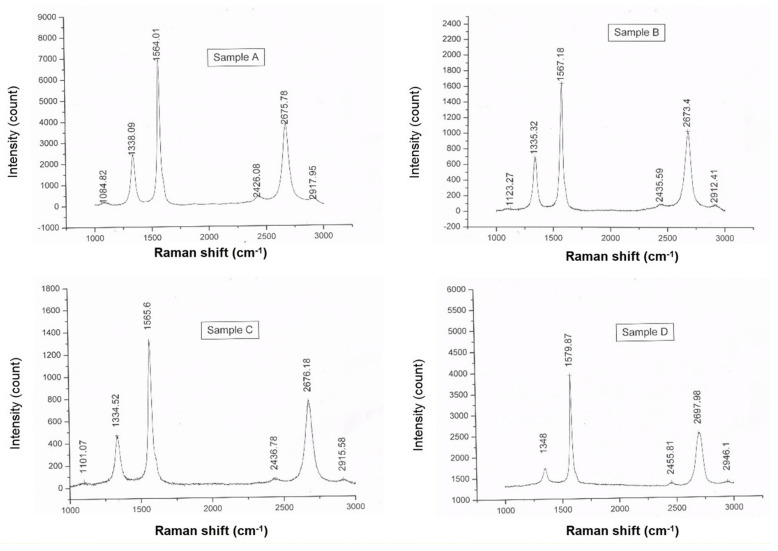
Raman spectra of (**A**) MWCNT-COOH, (**B**) MWCNT-CONOHME, (**C**) MWCNT-CON(OH)CO(Me), and (**D**) MWCNT-CON(OH)COPh.

Raman spectra were recorded for the MWCNT derivatives, as shown in Table 1 and Figure 3. All spectra exhibited peaks in ranges of 1334–1348 cm^−1^ (D band), 1564–1570 cm^−1^ (G band), and 2673–2682 cm^−1^ (2D band). The peaks visible at 1338, 1564, and 2675 cm^−1^ correspond to the D, G, and 2D bands of the functionalized starting material, MWCNTCOOH (A), as shown in Figure 3. The peaks appearing at 1335, 1567, and 2673 cm^−1^ correspond to the D, G, and 2D bands of MWCNT-CONOHMe (B), respectively, as shown in Figure 3B. The peaks at 1337, 1570, and 2682 cm^−1^ in Figure 3C correspond to the D, G, and 2D bands of MWCNT-CONOHCOMe (C), and those at 1334, 1565, and 2676 cm^−1^ in Figure 3D correspond to the D, G, and 2D bands of MWCNT-CONOHCOPh (D). Notably, not all the investigated compounds exhibited peaks in the range of 100–300 cm^−1^, which suggests a predictable lack of radial breathing mode (RBM) peaks for the MWCNTs in this region [56]. The D band is an indicator of chirality due to double resonance in the CNTs, and this band is used as a measure of disorder, as in graphite and small crystalline sp^2^ clusters [56]. The G band measures the graphitization of MWCNTs and corresponds to the tangent fluctuation of sp^2^ hybridization in carbon [56]. The D2 band is an indicator of the long-range graphite order due to the strong coupling between electrons and phonons [56].

A comparison of the D bands in the starting material, MWCNTCOOH (A), and the MWCNT-CONOHMe (B), MWCNT-CONOHCOMe (C), and MWCNT-CONOHCOPh (D) compounds reveals that the D bands of MWCNT-CONOHMe (B), MWCNT-CONOHCOMe (C), and D shifted to lower wavenumbers by 3, 2, and 4, cm^−1^, respectively. This was possibly caused by the increase in sp^2^-hybridized carbon in these functionalized products and the effect of electron withdrawal. Additionally, the intensities of the D bands of the MWCNT-CONOHMe (B) and MWCNT-CONOHCOPh (D) compounds were noted to decrease compared to that of compound MWCNTCOOH (A). On the other hand, the intensity of the D band of MWCNT-CONOHCOPh (D) was notably increased compared to that of compound MWCNTCOOH (A). The *I_D_*/*I_G_* ratios of the starting material, MWCNTCOOH (A), and the MWCNT-CONOHMe (B), MWCNT-CONOHCOMe (C), and MWCNT-CONOHCOPh (D) compounds were as follows: 0.36, 0.42, 0.44, and 0.37, respectively. This result reveals a slight increase in the *I*_D_/*I*_G_ of the products compared with that of the starting material, MWCNTCOOH (A), indicating an increase in sp^3^ due to the introduced functional groups in the MWCNTs.

### 3.3. X-ray Photoelectron Spectroscopy (XPS)

**Figure 4:**
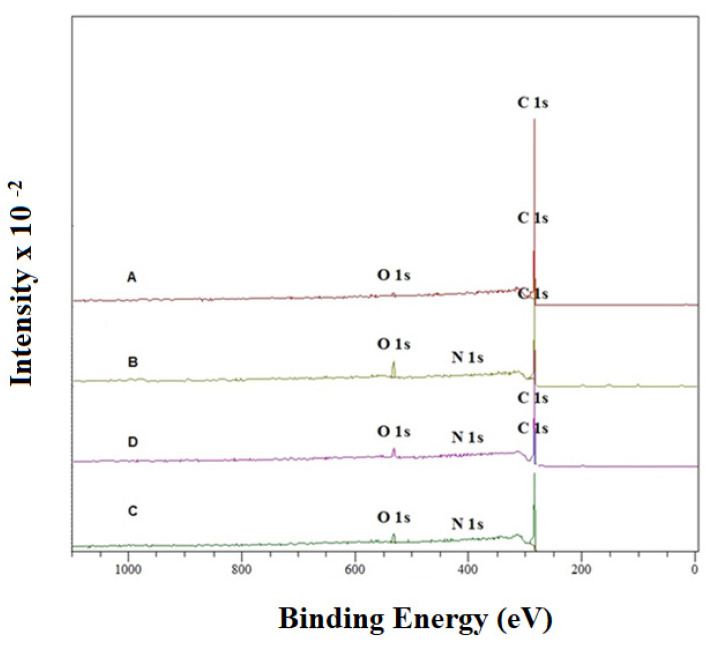
Wide-scan XPS of (**A**) MWCNT-COOH, (**B**) MWCNT-CONOHMe, (**C**) MWCNT-CONOHCOMe, and (**D**) MWCNT-CONOHCOPh.

**Figure 5:**
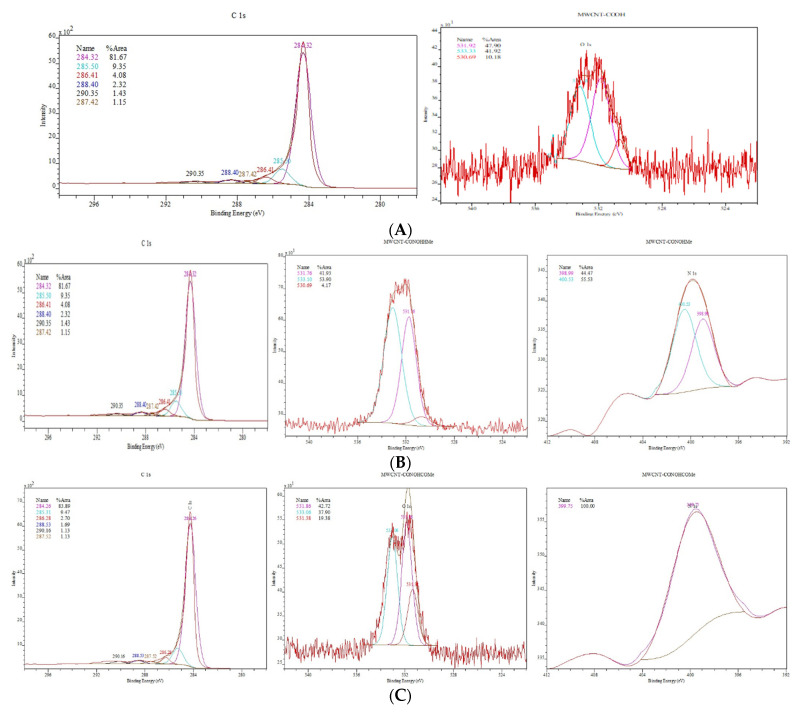

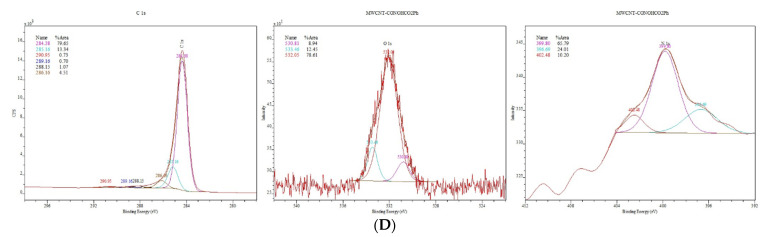
X-ray photoelectron spectroscopy (XPS) profiles of (**A**) MWCNT-COOH, (**B**) MWCNT-CONOHMe, (**C**) MWCNT-CONOHCOMe, and (**D**) MWCNT-CONOHCOPh.

X-ray photoelectron spectroscopy (XPS) was used to characterize the products and determine the functional groups in the MWCNTs, as shown in Figure 4. The transformations of MWCNT-COOH to the hydroxamic acid derivatives, B, C, and D, were confirmed with wide-scan XPS analysis and a comparison of the obtained results. Figure 5A shows the spectra of MWCNT-COOH with two binding energy peaks at 284.0 eV and 532 eV, which correspond to carbon and oxygen, respectively. Figure 5A–D shows the spectra of the compounds with hydroxamic acid derivatives B, C, and D, which display three peaks at 284.0, 532, and 400.0 eV, corresponding to carbon, oxygen, and nitrogen, respectively. The relative atomic concentrations of carbon and oxygen in A were 98.2792% and 1.72075%, respectively. The relative atomic concentrations in sample B were as follows: C, 92.3838%; N, 0.778012%; and O, 6.83822%. For samples C and D, the relative atomic concentrations were C, 95.1036 and 93.0865; N, 0.41164 and 1.04813; and O, 4.48464 and 5.86537, respectively. This indicates that functionalized MWCNTs containing hydroxamic acid of types B, C, and D, were successfully prepared.

The data obtained from the high-resolution XPS, as shown in Figure 4, indicated that the C1s spectrum of MWCNT-COOH (A) reveals a major peak at 284.4 eV (82.12%) was assigned to carbon atoms of C-C, a peak at 285.55 eV was assigned to C-H carbon, and a peak at 286.46 eV was assigned to C-OH (3.87%). The peak at 287.44 eV (1.10%), was ascribed to carbonyl C=O, and the peak at 288.97 eV was assigned to HO-C=O (1.70%). On the other hand, the O1s spectrum of MWCNT-COOH (A) was decomposed into two components at 531.76 eV (51.82%) and 533.17 eV(48.18%), which correspond to C=O and HO-C=O, respectively. The above results of the high-resolution XPS of MWCNT-COOH (A) were compared with results obtained from MWCNT-CONOHMe (B), MWCNT-CONOHCOMe (C), and MWCNT-CONOHCOPh (D). All the hydroxamic acid derivatives, B, C, and D, showed satisfactory characteristics in the high-resolution XPS spectra of the carbon region C1s, oxygen O1s region, and nitrogen N1s region [56].

### 3.4. Adsorption of Lead Ions

#### 3.4.1. Effect of Contact Time

**Figure 6:**
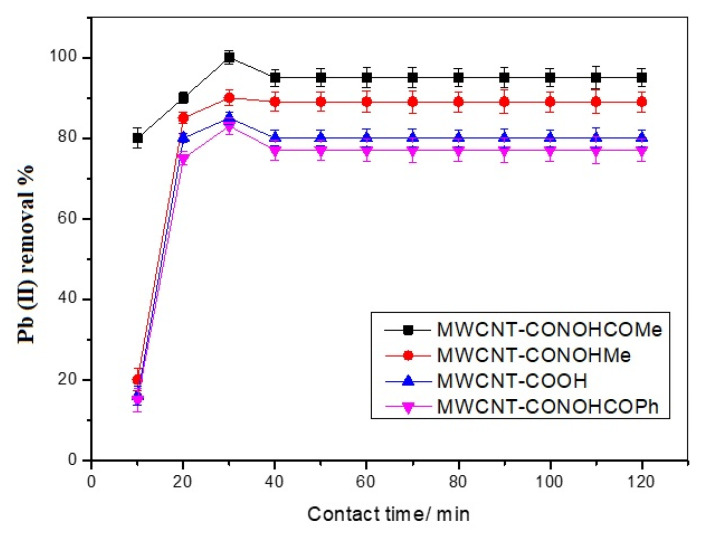
Effect of contact time on Pb(II) removal % using 10 mg of functionalized carbon nanotubes at pH 6 and 30 mg/L of Pb(II) at 30 °C.

Figure 6 shows the effect of contact time on Pb(II) removal efficiency using 10 mg of functionalized carbon nanotubes at a pH of 6 and 30 mg/L of Pb(II) at 30 °C. The data revealed that optimal removal is accomplished after ~50 min, approximately, for all four adsorbents; then, the adsorption stabilizes thereafter. The highly effective Pb(II) removal is primarily because of the presence of empty sites, which reduce in number consistently with time. Overall, phenomenal adsorption productivity concerning Pb(II) was exhibited by the four adsorbents in the following order: MWCNT-CONOHCOMe (**C**) > MWCNT-CONOHMe (B) > MWCNT-COOH (A) > MWCNT-CONOHCOPh (D). Amazingly, the Pb(II) removal percent with functionalized MWCNTs (B), (C), and (A) was greater than with MWCNT-CONOHCOPh (D). This could be due to the steric effect of the phenyl group, which reduces the number of binding sites on the produced MWCNTs [57].

#### 3.4.2. Effect of pH

**Figure 7:**
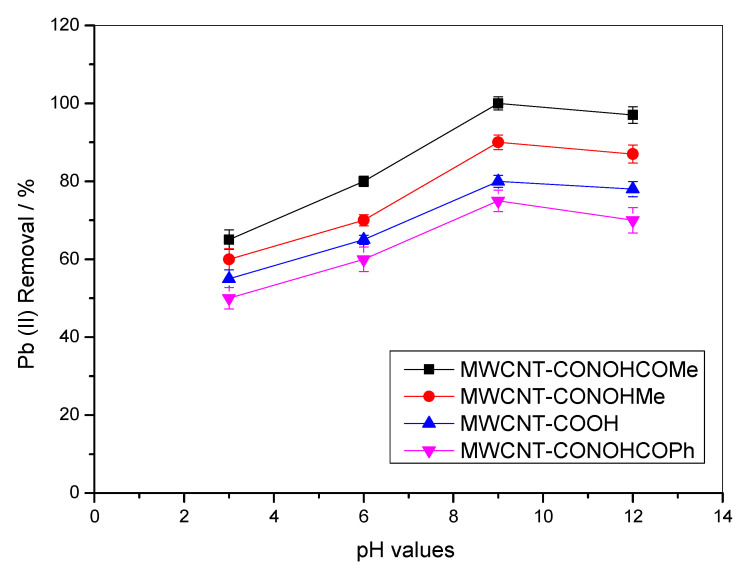
Effect of pH on Pb(II) removal % using 10 mg of functionalized carbon nanotubes and 30 mg/L of Pb(II) at 30 °C for 50 min.

Because it influences the solubility of metal ions, as well as the concentration of counter ions on the functional groups of the adsorbent and the degree of ionization in the adsorbate during the reaction, pH is also a significant parameter for the adsorption of metal ions from aqueous solutions. The change in the pH of the solution might affect the interaction between the charge on the adsorbent surface and the adsorbate. Therefore, the influence of solution pH on the adsorption efficiency of MWCNT–hydroxamic acid derivative Pb(II) was evaluated, as shown in Figure 7. The results showed that the functional groups produced by functionalization enhanced the ion exchange capabilities of carbon nanotubes, increasing the Pb^2+^ removal percentage by increasing pH values up to 8 and then decreasing them for all the MWCNT–hydroxamic acid derivatives. This was because of the chemical interaction between the Pb^2+^ ions and surface functional groups such as hydroxyl (–OH), carboxyl (–COOH), (-N-OH), and carbonyl (–C=O). However, MWVNT-CONOHCOMe showed a more dramatic increase due to the chemical interaction between the metal ions and surface functional groups such as hydroxyl (–OH), carboxyl (–COOH), (-N-OH), and carbonyl (–C=O)) [58].

#### 3.4.3. Competitive Adsorption

**Figure 8:**
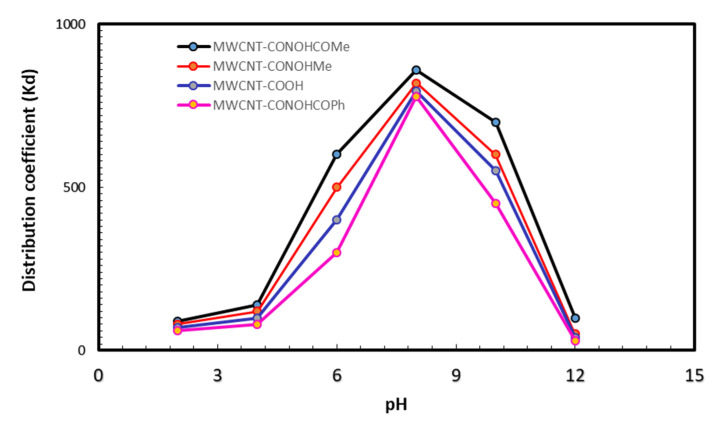
Effect of pH on the distribution coefficient for Pb(II) adsorbed onto different prepared adsorbent materials.

In essence, the distribution coefficient (*K_d_*) reflects the actual performance of an adsorbent according to the ratio of the adsorbent’s adsorption capacity to the liquid’s final concentration of adsorbate at equilibrium [49,50], while the operating conditions can affect the adsorption capacity and removal efficiency, as shown in Table 2. The effectiveness of the prepared adsorbent materials was evaluated using the distribution coefficient (*K_d_*) according to Equation (5). Figure 8 shows that increasing pH caused more metal ions to be accessible to migrate from the solution to the surface of the prepared adsorbent materials, which resulted in an increase in *K_d_* values for Pb(II). A comparison of the distribution coefficient values with others from previous studies is illustrated in Table 3 [59,60,61,62,63,64,65].

#### 3.4.4. Effect of Initial Concentration of Lead

**Figure 9:**
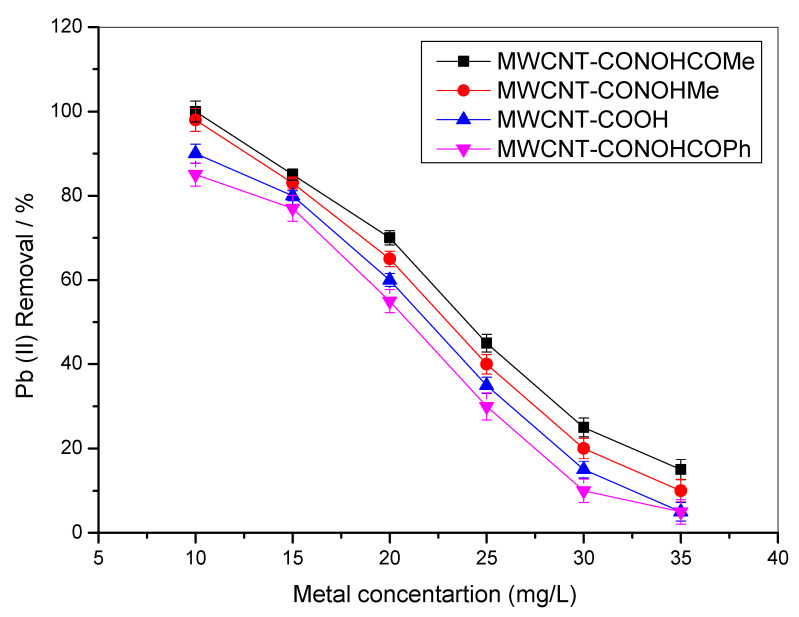
Effect of initial concentration of Pb(II) on Pb(II) removal % using 10 mg functionalized carbon nanotubes and a pH of 8.0 for 50 min at 30 °C.

The effects of the initial concentration of Pb(II) on the removal percent are shown in Figure 9. The optimal Pb(II) removal percent by the four adsorbents was observed to correspond to a concentration of 10 mg. The results showed that at low concentrations, the proportion of the metal ions to the available sorption sites was low, and accordingly, adsorption under these conditions is free of the underlying fixation. However, as the concentration of Pb(II) increases, the sites accessible for adsorption decrease in number, and consequently, the removal percent of Pb(II) is profoundly affected by the underlying concentrations. The results showed that the pace of lead ion expulsion diminishes as the underlying centralization of metal ions increases.

#### 3.4.5. Effect of Functionalized MWCNTs Dose

**Figure 10:**
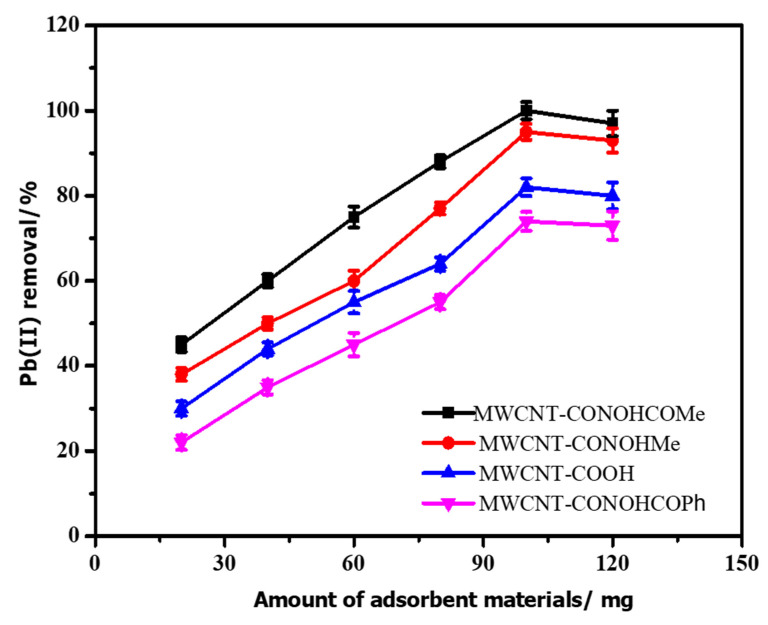
The effect of adsorbent material doses on Pb^2+^ removal % using a pH of 8.0 for 50 min at 30 °C.

Six weights of MWCNT-CONOHCOMe (C) (20, 40, 60, 80, 100, and 120 mg) were employed to adsorb Pb^2+^ ions from an aqueous solution to elucidate the role of adsorbent material doses on the Pb^2+^ removal percent, as shown in Figure 10. The data showed that the removal percent increases by increasing the adsorbent material doses up to 100 mg and then becomes approximately constant due to an increase in the adsorbent surface, which increased the availability of adsorption sites. Furthermore, no further increase in the removal percent was observed using adsorbent doses of more than 100 mg. This finding demonstrated that the metal ions were first adsorbed externally, and the adsorption was fast. When the adsorbent’s exterior surface was saturated, the metal ions adsorbed into the porous structure and eventually reached an approximately constant value, where no further adsorption happened [66]. The Pb^2+^ removal percent for MWCNT-CONOHCOMe, MWCNT-CONOHMe, MWCNT-COOH, and MWCNT-CONOHCOPh was 100%, 96%, 95%, and 74%, respectively.

#### 3.4.6. Equilibrium Isotherm

**Figure 11:**
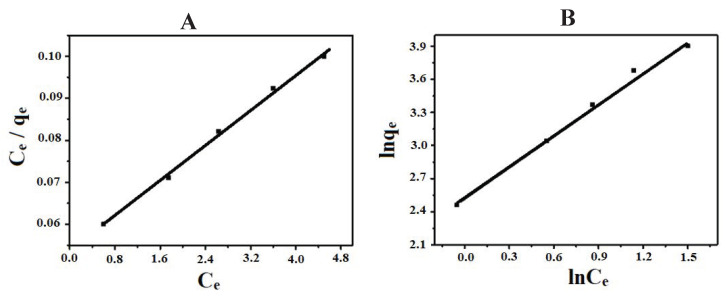
Langmuir and Freundlich isotherm models for the adsorption of lead onto (**A**) and (**B**) (adsorbent dose, 0.01 mg/mL).

The relationship between the oxidized MWCNTs and MWCNTs functionalized with hydroxamic acid derivatives with the corresponding amount of adsorbate was examined using Freundlich and Langmuir isotherm models and their equilibrium concentration in solutions [58,66]. Figure 11 and Table 4 show Langmuir and Freundlich isotherm models for the adsorption of lead onto selected MWCNT-CONOHCOMe with a dose of 0.01 mg/mL. The boundaries of the Freundlich and Langmuir adsorption isotherms were determined using the optimal condition parameters obtained in the trial part of the experiment.

Figure 11 shows Langmuir and Freundlich isotherm models for the adsorption of lead ions using an adsorbent dose of 0.01 mg/mL. Table 4 shows the correlation coefficient value in the Langmuir model (R^2^), which is equal to 1 and is higher than the correlation coefficient value in the Freundlich model (R^2^), which is equal to 0.736, and the maximum adsorption capacity of the adsorbent (q_max_), 512 mg/g. The high correlation coefficient (R^2^ = 1) indicates the suitability of the Langmuir adsorption model, and the data demonstrated that the adsorption investigated in this study does not fit the Freundlich model. In conclusion, the Langmuir model best represented the adsorption data, implying that Pb(II) ion adsorption is monolayer-reporting, with the maximum value, q_max_, of Pb(II) ion adsorption on MWCNT-CONOHCOMe being 512 mg/g.

#### 3.4.7. Adsorption Kinetics

To investigate the kinetics and mechanism of adsorption, the pseudo-first-order and pseudo-second-order models were considered for the experimental data:

Pseudo-first-order kinetics mode [54,55]:

(13) $$\ln(q_{e}-q_{t}^{})=\ln(q_{e})-k_{1}t$$

Pseudo-second-order model [53]:

(14) $$\frac{t}{q}=\frac{1}{K_{2}q_{e}^{2}}+\frac{t}{q_{e}}$$

where *q_e_* (mg/g) and *q_t_* (mg/g) are the amounts of Pb(II) at time *t*, *k*_1_ (1/min) is the pseudo-first-order rate constant, and *t* represents time. *k*_2_ (g/mg/min) is the rate constant of the pseudo-second-order equation [57,58]. Table 5 shows the adsorption parameters obtained using the pseudo-first-order and pseudo-second-order models for Pb(II) on oxidized MWCNTs and MWCNTs functionalized with hydroxamic acid derivatives. The obtained data reveal that the pseudo-second-order model appropriately represents the adsorption of Pb(II) on MWCNT-COOH (A), MWCNT-CONOHMe (B), MWCNT-CONOHCOMe (C), and MWCNT-CONOHCOPh (D). The correlation coefficients (*R*^2^) of the pseudo-second-order model are noted to be 0.988, 0.978, 1, and 0.999 for MWCNT-COOH (A), MWCNT-CONOHMe (B), MWCNT-CONOHCOMe (C), and MWCNT-CONOHCOPh (D) respectively, while those of the pseudo-first-order model are 0.40, 0.35, 0.62, and 0.33, respectively. Additionally, the experimental adsorption limits (*q_e_*_,exp_) of MWCNT-COOH (A), MWCNT-CONOHMe (B), MWCNT-CONOHCOMe (C), and MWCNT-CONOHCOPh (D) are 9.63, 7.68, 9.55, and 7.5 mg/g respectively, which are in good agreement with the adsorption limits (*q_e_*_,cal_) evaluated using the pseudo-second-order model (9, 80, 8.2, 9.77, and 6.69 mg/g, respectively).

#### 3.4.8. Adsorption Thermodynamics

Thermodynamic parameters such as Gibbs free energy (Δ*G*), enthalpy (Δ*H*), and entropy (Δ*S*) were evaluated [57,58] at 298K, as shown in Table 6. The negative values of Δ*G* demonstrate that the adsorption of Pb(II) onto MWCTN is a spontaneous process and the value of Δ*G* increases as temperature increases. Therefore, the efficiency of adsorption is more effective in higher temperatures. The positive values of Δ*S* indicate the endothermic nature of the adsorption, whereas the positive values of Δ*H* indicate a haphazardness in growing in the strong fluid interface during the adsorption system. Therefore, the adsorption of Pb(II) on the fabricated materials was of an endothermic, disordered, and unconstrained nature.

#### 3.4.9. Intraparticle Diffusion Model

The intraparticle diffusion model, which reflects pore diffusion, was established and planned by Weber and Morris (1963) [67]. A plot of adsorbate uptake vs. the square root of time (t^0.5^) is based on the following equation:

(15) $$q_{t}=K_{i}t^{\frac{1}{2}}+C$$

*C* is an arbitrary constant demonstrating the thickness of the boundary layer, and a greater value of *C* characterizes a thicker boundary layer [68]. If the value of *C* is zero, which corresponds to no boundary layer, the linear line should pass through the origin. The internal diffusion model assumes that the internal diffusion of the adsorbate is the slowest step, resulting in the rate-controlling step through the adsorption route, and the adsorption is instantaneous [60]. Consequently, film diffusion could be overlooked due to no or less thickness; accordingly, intraparticle diffusion would continue the rate-controlling step through the whole adsorption kinetic route. Many studies have reported nonzero intercepts, indicating that the rate-limiting step involves both intraparticle and film diffusion in most adsorption processes [42,69]. In this study, due to the very low *R*^2^ with the single regression line, we were unable to predict one linear line for the experimentation results. Consequently, the outcomes were separated with two linear regressions, demonstrating that both film and intraparticle diffusion control the adsorption diffusion. The initial rapid increase was also represented by film diffusion involved in the adsorption of lead [42].

### 3.5. Comparative Study

The literature on lead adsorption using carbon nanotubes is restricted to a few cases, as indicated in Table 7. CNTs are limited in their uses due to their low solubility in most solvents. The materials’ low solubility in aqueous and organic solvents, as well as their restricted compatibility with polymer matrices, are important disadvantages that prevent them from reaching their full potential. Table 7 shows that the functionalized MWCNTs containing different hydroxamic acid derivatives influenced a greater removal percent for Pb^2+^ than the described adsorbents. The stated removal percent showed that the obtained functionalized MWCNTs nanocomposites could be more efficient than the other adsorbents.

## 4. Conclusions

Multi-walled carbon nanotubes (MWCNTs) functionalized with four novel hydroxamic acid derivatives, MWCNT-COOH (A), MWCNT-CONOHMe (B), MWCNT-CONOHCOMe(C), and MWCNT-CONOHPh (D), were developed in this study and applied for the elimination of Pb(II) ions from water. The prepared samples were characterized using various techniques, such as scanning electron microscopy, Raman spectroscopy, and X-ray photoelectron spectroscopy. The investigation of a few SEM images of the functionalized MWCNTs facilitated the measurement of the solid length of a couple of nanotubes, and all of them were in the range of 19–31 nm. The Raman results showed the *I*_D_/*I*_G_ ratios of the starting material, MWCNTCOOH (A), and the MWCNT-CONOHMe (B), MWCNT-CONOHCOMe (C), and MWCNT-CONOHCOPh (D) compounds were as follows: 0.36, 0.42, 0.44, and 0.37 respectively. This result revealed a slight increase in the *I*_D_/*I*_G_ of the products compared with that of the starting material, MWCNTCOOH (A), indicating an increase in sp^3^ due to the introduced functional groups in the MWCNTs. Moreover, XPS data indicated that functionalized MWCNTs containing hydroxamic acid derivatives were successfully prepared. The effectiveness of the prepared compounds to remove Pb(II) was considered to depend on the adsorbent dose, pH, initial concentration of Pb(II) ions, and contact time. The four adsorbents showed remarkable effectiveness toward Pb(II), with a quantitative percentage of removal. The optimal adsorption effectiveness was found to be at room temperature at contact for 50 min, 8 pH, and a Pb^2+^ initial concentration of 10 mg, and the adsorption capacity was 140 mg·g^−1^ for the MWCNT-CONOHCOMe. An investigation of adsorption kinetics revealed that the adsorption of Pb(II) by the four adsorbents followed pseudo-second-order and Langmuir isotherm models. The thermodynamic investigation indicated negative free energy, which revealed unconstrained adsorption at room temperature.

## Figures and Tables

**Table 1:** Raman shifts (cm^−1^) of (A) MWCNT-COOH, (B) MWCNT-CONOHMe, (C) MWCNT-CONOHCOMe, and (D) MWCNT-CONOHCOPh.

Sample		*D Band* (cm^−1^)	*G Band* (cm^−1^)	G* (cm^−1^)	*2D* (cm^−1^)	*D + G* (cm^−1^)	*I_D_/I_G_*
A	1084.82	1338.09	1564.00	2426.08	2675.78	2917.95	0.36
B	1123.26	1335.32	1567.18	2435.59	2673.40	2912.14	0.42
C	1101.07	1334.52	1565.60	2436.78	2676.18	2915.58	0.44
D	-	1348.00	1579.87	2455.81	2697.98	2946.10	0.37

**Table 2:** The prepared adsorbent materials’ performance evaluations for removing Pb(II) at 8 pH and a 100 mgL^−1^ metal starting concentration.

Adsorbent Materials	Maximum Removal Efficiency (%)	Distribution Coefficient (L g^−1^)
MWCNT-CONOHCOMe	100	860
MWCNT-CONOHMe	96	824
MWCNT-COOH	88	794
MWCNT-CONOHCOPh	72	770

**Table 3:** A review of the performance of some carbon-based adsorbent materials used to remove Pb(II) from water.

Adsorbent Nanomaterials	Maximum Adsorption Capacity (mg g^−1^)	Distribution Coefficient (L g^−1^)	Reference
Chitosan-pyromellitic dianhydride modified biochar (CPMB)	9.24	0.02	[59]
Hydrated manganese oxide biochar nanocomposite (HMO-BC)	67.9	1.20	[60]
Magnetic graphene composite material (Fe_3_O_4_-GS)	27.95	47.0	[61]
poly3- aminopropyltriethoxysilane oligomer-linked graphene oxide composite (PAS-GO)	312.5	3.60	[62]
Magnetic ethylene diamine-functionalized graphene oxide (MDFGO)	145	73	[63]
Magnetic graphene oxide/MgAl-layered double hydroxide nanocomposite	192.31	0.6	[64]
Sulfanilic acid-functionalized graphene oxide (SGO) Reduced sulfonated graphene oxide (rSGO) Graphene oxide (GO) Reduced graphene oxide (rGO)	454.54 400 303.03 140.84	38 29 17 6	[65]
MWCNT-CONOHCOMe MWCNT-CONOHMe MWCNT-COOOH MWCNT-CONOHCOPh	512 504 490 468	860 820 796 770	This work This work This work This work

**Table 4:** Langmuir and Freundlich parameters for Pb(II) adsorption onto MWCNT-CONOHCOMe.

	Langmuir Adsorption Isotherm				Freundlich Adsorption Isotherm		
	*q_m_* (mg/g)	*k_L_* (L/mg)	*R* ^2^	*R_L_*	*K_F_* (mg/g)	1/*n_F_*	*R* ^2^
MWCNT-CONOHCOMe	512	0.188	0.998	0.261	210	2.512	0.736

**Table 5:** Parameters were evaluated using the pseudo-first-order and pseudo-second-order kinetic models.

Prepared Samples	Pseudo-First-Order			Pseudo-Second-Order			
	*k*_1_ (_h_^−1^)	*R* ^2^	*q_e_*_,cal_ (mg/g)	*K_2_* (g/mg·h)	*R* ^2^	*q_e_*_,cal_ (mg/g)	*q_e_*_,exp_ (mg/g)
A	0.44	0.40	1.66	0.10	0.988	9.80	9.63
B	0.50	0.35	1.30	0.12	0.978	8.2	7.68
C	0.45	0.62	1.65	0.10	0.999	9.77	9.55
D	0.91	0.33	1.04	0.14	0.887	6.69	7.5

**Table 6:** Thermodynamic parameters of adsorption of 100 mg L^−1^ Pb(II) onto A, B, C, and D.

	Δ*H* (kJ mol^−1^)	Δ*S* (J mol^−1^ K^−1^)	Δ*G* (kJ mol^−1^)
A	3.50338	52.1216	−15,636.6
B	2.0560	46.11	−13,105.2
C	1.726	22.06	−10,114.4
D	0.5060	19.11	−9690.7

(A) MWCNT-COOH, (B) MWCNT-CONOHMe, (C) MWCNT-CONOHCOMe, and (D) MWCNT-CONOHCOPh at 298 K.

**Table 7:** Some CNT adsorbent capacities have been reported for lead ion adsorption.

Type of Adsorbents	Adsorption Capacity (mg/g)	pH	Ref.
CNTs	17.44	7	[70]
MWCNTs	78.7	7	[43]
Oxidized MWCNTs	93	7	[71]
CNTs/Al_2_O_3_	67.5	7	[72]
MnO_2_/CNTs	76	7	[36]
MWCNT-COOH	480	8	This study
MWCNT-CONOHMe	504	8	This study
MWCNT-CONOHCOMe	512	8	This study
MWCNT-CONOHCOPh	468	8	This study

## Data Availability

The authors confirm that the data of this study are available within the article. Raw data are available from the corresponding author upon request.
